# Determination of the Same Cardiac Cycle When Analyzing Cine Clips for Use by FetalHQ to Assess Intraobserver and Interobserver Variability. Comment on Mlodawski et al. Reproducibility Challenges in Fetal Cardiac Function Analysis with 2D Speckle-Tracking Echocardiography: Insights from FetalHQ. *J. Clin. Med.* 2025, *14*, 3301

**DOI:** 10.3390/jcm15072468

**Published:** 2026-03-24

**Authors:** Greggory R. DeVore

**Affiliations:** Fetal Diagnostic Centers, Suite 330, 50 Alessandro Place, Pasadena, CA 91105, USA; grdevore@gmail.com; Tel.: +1-626-840-6988

## 1. Introduction

I read with interest the study by Mlodawski et al. entitled, “Reproducibility Challenges in Fetal Cardiac Function Analysis with 2D Speckle-Tracking Echocardiography: Insights. from FetalHQ” [1]. While the authors describe their technique for making measurements of the length and width of the four-chamber view and identifying the cardiac cycle for fetalHQ measurements of the ventricles, there is an important concept when determining the intraobserver and interobserver variability which the authors apparently did not follow by examining the same cine loop but not the same cardiac cycle. The principles the authors should have followed are listed as follows:

## 2. Measuring the Four-Chamber View

To determine the intraobserver and interobserver variability the authors should identify an end-diastolic image of the four-chamber view which was used as the source for the end-diastolic length and width measurements for assessing both the intraobserver and interobserver variability. This approach would negate the variable of choosing a different end-diastolic image from the same cine clip for their analysis. In addition, it would provide more precise data that would focus on the same examiner making the measurements at different times on the same end-diastolic image as well as two examiners making the measurements from the same image.

## 3. Ventricular FetalHQ Measurements

The authors should use the same cardiac cycle derived from the M-mode technique described in their paper. By doing so, the authors can eliminate the effect of changes in the 4-chamber view image as the result of different cycle cycles from the same cine clip. Once the cardiac cycle is selected, the authors could determine the intraobserver and interobserver variability for the same image. In addition, if the authors have QUIVER [2] on their system, this would assist the authors in identifying the endocardial border of the right and left ventricles. The benefit of quiver is that it is a real-time image of the four-chamber view that loops 2 frames before and 2 frames after the selected clip. This allows the examiner to more clearly identify the endocardial borders. Using the same cardiac cycle for the analysis would negate any effect of the authors selecting different cardiac cycles from the cine clip.

In conclusion, the authors need to clarify if they followed the above approach or simply used different cardiac cycles from the same cine clip from the patients studied. If the latter is the case, then the variable of selecting different cardiac cycles from the same cine clip may compromise the analysis since the purpose of the study is to determine the expertise of the same examiner as well as two examiners in making the measurements.
